# Calculating indirect costs from international PEPFAR implementing partners

**DOI:** 10.1371/journal.pone.0206425

**Published:** 2018-10-29

**Authors:** Brian Honermann, Alana Sharp, Jennifer Sherwood, Pratima Kshetry, Austin Jones, Richael O’Hagan, Laura Lazar, Christina Chandra, Topher Hoffmann, Greg Millett

**Affiliations:** amfAR, the Foundation for AIDS Research, Washington, DC, United States; NPMS-HHC CIC / LSH&TM, UNITED KINGDOM

## Abstract

**Background:**

UNAIDS estimates global HIV investment needs in low- and middle-income countries (LMICs) at $26 billion per year in 2020. Yet international financing for HIV programs has stagnated amidst despite the increasing number of people requiring and accessing treatment. Despite increased efficiencies in HIV service delivery, evaluating programs for greater efficiencies remains necessary. While HIV budgets have been under scrutiny in recent years, indirect costs have not been quantified for any major global HIV program, but may constitute an additional avenue to identify program efficiencies. This analysis presents a method for estimating indirect costs in the President’s Emergency Plan for AIDS Relief (PEPFAR).

**Methods:**

Utilizing PEPFAR country operational plan (COP) funding data from 2007 to 2016 for international organizations (IOs) and universities and standard regulatory cost bases, we calculated modified total direct costs on which indirect cost rates may be applied by partner and funding agency. We then apply a series of plausible indirect cost rates (10%–36.28%) to develop a range for total indirect costs that have accrued over the period.

**Findings:**

Of $37.01 billion in total COP funding between 2007 and 2016, $22.24 billion (60.08%) was identifiably allocated to IOs ($17.95B) and universities ($4.29B). After excluding funding for sub-awards ($1.92B) and other expenses ($3.89B) to which indirect rates cannot be applied, $16.44B remained in combined direct and indirect costs. From this, we estimate that between $1.85B (8.30% of total international partner funding) and $4.34B (19.51%) has been spent on indirect costs from 2007–2016, including $157-$369 million in 2016.

**Interpretation:**

To our knowledge, this is the first analysis to quantify the indirect costs of major implementing partners of a global HIV funder. However, lack of transparency in the indirect cost rates of non-University international partners creates an opaque layer of programmatic costs. Given the current funding environment and evolution of HIV programming in PEPFAR countries, the findings motivate a re-examination of the current policies and the return on investment in indirect cost recovery across the PEPFAR program.

## Introduction

The UNAIDS Fast Track agenda and 90-90-90 targets have galvanized the world to re-focus on the essential priorities for combating HIV globally. UNAIDS estimates that achieving the 90-90-90 goals will require an annual commitment of $26.2 billion for the epidemic in low- and middle-income countries, up from $19.2 billion in 2014.[1] However, international donor funding for HIV programs in low- and middle-income countries (LMIC) has declined for two consecutive years.[2] Combined U.S. contributions to the U.S. President’s Emergency Plan for AIDS Relief (PEPFAR) and the Global Fund have been essentially flat since 2009 and continue to be threatened with budget cuts by the U.S. government.[3] These resource constraints coincide with welcomed increases in life-expectancy of people living with HIV (PLHIV) as more people receive treatment and implementation of test and treat for individuals being newly identified.[4]

While advocacy for additional resources is still greatly needed, greater efficiencies in HIV funding must also be found if 90-90-90 is to be reached under these conditions. Scaling up antiretroviral treatment programs since 2003 has resulted in vast increases in knowledge about implementing programs in low-resource settings and enormous improvements in the delivery of treatment. Drug prices have declined 99% since 2000,[5] overall treatment costs have declined,[6] and health system efficiency at delivering services has vastly improved. Even lower drug prices are on the horizon with the introduction of dolutegravir-based regimens capped at just $75 USD per patient per year.[7] Implementation of differentiated care models are also likely to realize greater efficiencies.[8] However, the world cannot rely on declining drug prices to meet the HIV funding deficit. All tenable options for increasing the efficiency and impact of waning international resources must be explored.

One area for potential efficiency improvement is the proportion of HIV funding reserved for the indirect costs of major implementing partners. Indirect costs are general costs of administering and running programs that support organizational needs beyond a single grant. Examples include costs associated with buildings, maintenance, depreciation, or administrative staff.[9, 10] While there is broad recognition of the need for funders to compensate organizations for such costs, they have also long created controversy due to concerns about equal treatment of local and international partners, the overall rates that are being charged, and perceptions of effectiveness.[11–14]

Central to this debate is the issue of financial transparency and careful consideration of the relationship between indirect costs and efficient and effective management of programs. In the case of grants by the United States Federal government, indirect cost recovery is determined through private Negotiated Indirect Cost Rate Agreements (NICRAs) established between the U.S. Government (USG) and each organization. PEPFAR has taken large strides in making more financial and programmatic data available in recent years—including very recent releases of anonymized partner performance and site level data.[15–18] Yet the lack of transparency in indirect costs or rates have made both evaluations of the effectiveness of indirect costs, and organizational accountability for these rates, difficult.

To our knowledge, total funding for indirect costs has never been quantified for any major HIV program, constituting a gap in the HIV global financing discussion. Our analysis develops a method to estimate the range of indirect costs that have accrued to PEPFAR between 2007 and 2016 by international partners in an effort to better quantify the levels of funding used for indirect costs.

## Methods

Organizations wishing to claim indirect cost reimbursement from USG may negotiate a NICRA with a cognizant agency—the agency through which the largest portion of its federal funding is sourced.[19] While there are separate guidelines for non-profit organizations,[9] educational institutions,[10] and commercial entities,[20] the overarching principles are the same. In the case of PEPFAR partners, the cognizant agency is typically the Department of Health and Human Services (HHS) or the United States Agency for International Development (USAID). Foreign organizations can apply for a NICRA through the in-country mission.[19] The Office of the Global AIDS Coordinator (OGAC) that leads PEPFAR—housed within the State Department—is not part of the process of negotiating NICRAs. Once established, NICRAs are applicable across the federal government for the period covered and may be considered as provisional rates subject to renegotiation beyond the period. Organizations that do not have an established NICRA are able to claim a *de minimus* 10% indirect cost rate or are required to direct bill any such costs.[19]

Of note, NICRAs generally contain two or three distinct rate agreements: indirect cost rates (which may be split between “Overhead” and “General and Administrative” (G&A)) and fringe benefit rates. The latter are rates applied to salaries and wages for employees directly engaged on a project to cover costs associated with health insurance, vacation time, or retirement benefits and are included as direct costs of grants. Our analysis only relates to the indirect cost rates. Overhead and G&A rates compensate for different components of what may generally be considered indirect costs and may have different cost bases depending on organizational structure. For the purposes of this analysis, Overhead and G&A are combined into general indirect cost rates.

Indirect costs are calculated by multiplying the NICRA’s indirect cost rate by the Modified Total Direct Cost (MTDC) of each contract or grant. Federal regulations define MTDC as including direct salaries and wages, applicable fringe benefits, materials and supplies, services, travel, and the first $25,000 of each sub-award.[21] MTDC explicitly excludes:

- Sub-award amounts greater than $25,000;
- Capital expenditures;
- Equipment;
- Rental Costs; and
- Several categories of expenses not relevant to PEPFAR programming.

We have additionally identified several examples of ARV commodity procurement being excluded from MTDC in NICRAs or agreements.[22] For this reason, we have additionally excluded ARV procurement from MTDC in this analysis for all partners.

Individual NICRAs can negotiate different cost bases on which indirect cost rates will be charged. For instance, a partner may negotiate only to have indirect cost rates applied to salaries, wages, and fringe benefits rather than full MTDC. Since the rate in such cases would typically be higher than when indirect costs are applied to the full MTDC amount, we assume that the total indirect costs in either scenario would be approximately equal.

### Calculating indirect costs

**Fig 1:**
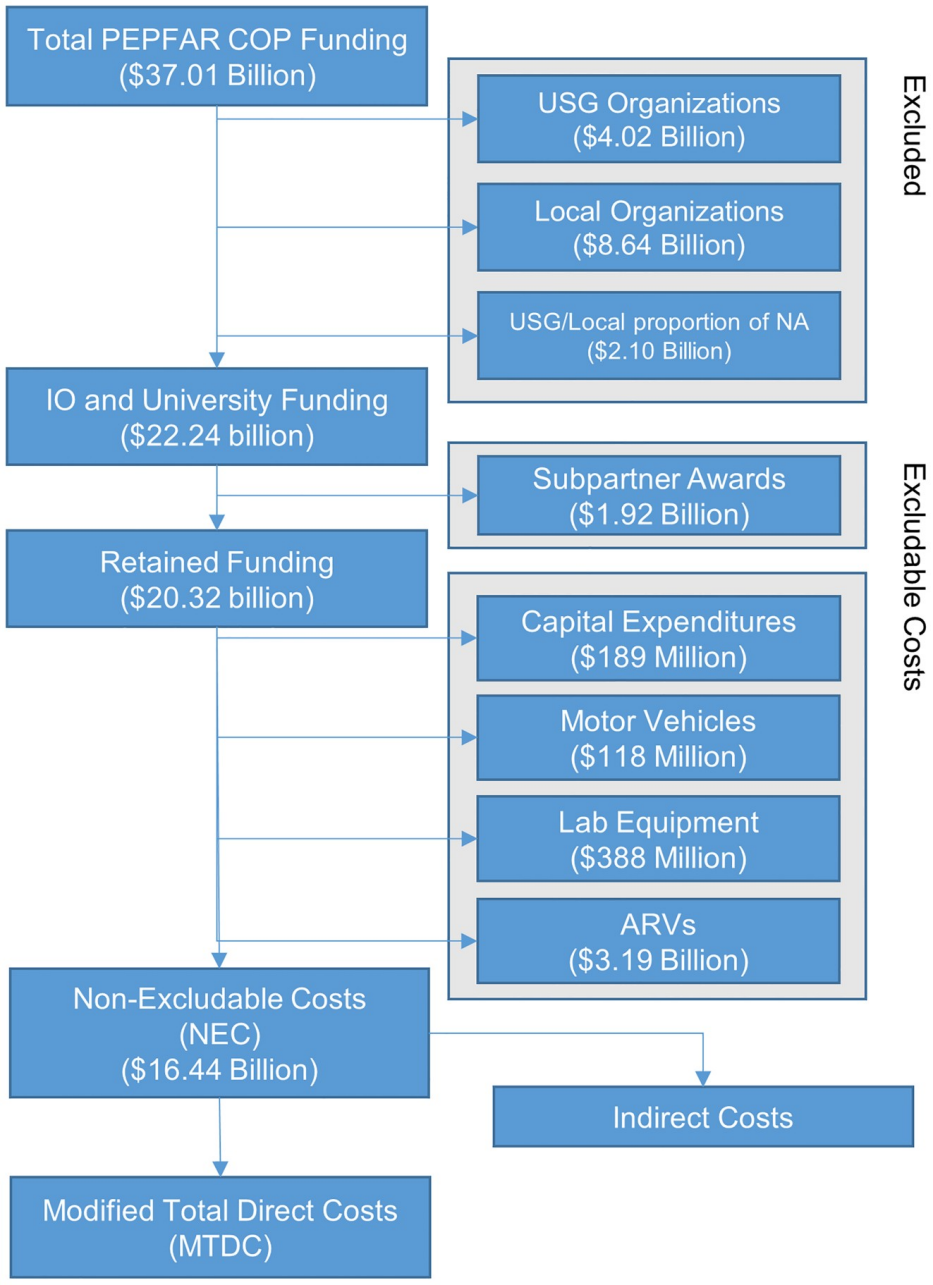
Calculating modified total direct costs and indirect costs. COP: Country and Regional Operational Plans; USG: United States Government; NA: Not Available; IO: International Organization; ARVs: antiretroviral medications; NEC: combined modified total direct costs (MTDC) and indirect costs.

Publicly available funding data from PEPFAR are limited to funding levels for different programmatic areas and do not disaggregate direct and indirect costs. To estimate indirect costs, the following process of elimination is used. First, funding for costs excluded from MTDC in the form of sub-awards, capital expenditures, and equipment are subtracted from each grant. The remaining total (the ‘non-excludable costs,’ or NEC) is therefore equal to the sum of the MTDC and indirect costs. By applying an indirect costs rate to this sum, the proportion that is indirect costs can be estimated. This method of calculating indirect costs (on a per-partner, per-agency, per-year basis) is shown by the following identities (Fig 1):

$${Total}_{ijt}={Sub}_{ijt}+{Ret}_{ijt}$$

$${Ret}_{ijt}={EC}_{ijt}+{NEC}_{ijt}$$

$${NEC}_{ijt}={MTDC}_{ijt}+{Ind}_{ijt}$$

$${Ind}_{ijt}=r_{ijt}{MTDC}_{ijt}$$

Where:

- *i* = Partner
- *j* = Funding Agency
- *t* = Year
- *Total* = Total allocated to partner
- *Sub* = Total distributed as sub-awards
- *Ret* = Total retained by partner after sub-awards
- *EC* = Excludable costs
- *NEC* = Non-excludable costs
- *MTDC* = Modified total direct costs
- *Ind* = Indirect costs
- *r* = Indirect cost rate

### Total funding (Total_ijt_)

PEPFAR publishes funding allocated to partners in Country Operational Plan (COP) documents on an annual basis. COP documents for years 2007–2016 were downloaded from PEPFAR’s website and the data extracted.[15] These data are available through amfAR’s online COPs database.[18] Funding was characterized across 8 categories: 1) Country; 2) Year; 3) Mechanism Number; 4) Implementing Partner; 5) Organizational Type of Partner; 6) US Government (USG) Funding Agency; 7) Budget Code; and 8) Planned Funding Amount. Additional mechanism-specific data on sub-awards and cross-cutting funding categories were also extracted. Where total planned funding associated with mechanisms was less than the total national funding, the difference was categorized as “Not Available (NA)” by country, year, and budget code. NA amounts exist primarily because not all agreements are awarded by the time COPs are published.

All unique implementing partners identified as receiving at least $1 in COP funding in any year (n = 828) were classified as “International”, if the organization is registered outside of PEPFAR program countries (this includes both universities and other international organizations (IOs)), “Local”, if the partner is based in a PEPFAR program country, “USG”, if the partner is a United States Government agency, or “NA”, if the location or type could not be determined. Two coders classified each implementing partner and discrepancies were assessed by a third coder.

Total_ijt_ is then calculated as the sum of total funding by partner, funding agency, and year. NA amounts are allocated to International, Local, and USG partners proportionately. USG and Local organizations are then excluded from the model. Management costs for USG are not part of our analysis. While Local organizations are eligible to apply for NICRAs, the frequency of country-based partners negotiating such rates is not known.

### Sub-award exclusions (*Sub*_*ijt*_)

As noted above, MTDC excludes sub-award amounts greater than $25,000. In COP documents prior to 2010, both primary and sub-award funding totals were released, but were not publicly available after 2010. We used funding data from the years with available sub-award information to estimate the proportion of grant totals not disbursed in sub-awards and that is retained by the partners (hereafter retention rate, *Ret*). To our knowledge, no major policy changes occurred between 2010 and 2016 that would substantially affect these retention rates. Because retention rates may vary by funding agency, partner retention rates were calculated by funding agency.

Additionally, as sub-awards to IOs and Universities have indirect cost rates applied by the sub-awardees, we adjusted partner retention rates for the proportion of sub-award funding that goes to other IOs or Universities, creating an “applied retention rate". To estimate the proportion of sub-awards to other IOs and Universities, we categorized all unique sub-partners (n = 1,899) awarded more than $100,000 within a single agency (n = 515) in 2007 by international location. Sub-partners awarded less than $100,000 were treated as local, as were sub-partners categorized as NA. These amounts were then used to calculate the proportion of sub-award funding directed to Local vs. International partners by agency.

For all partners without sub-award funding data between 2007 and 2009, we applied an agency level retention rate. To calculate agency retention rates, we calculated the percent of total funding retained by partners by agency, limiting to partners that reported sub-award data (N = 97; 34.28%). For agencies with no IOs or Universities with sub-award data between 2007 and 2009, we applied a total funding average.

We do not adjust for the first $25,000 of each sub-award on which indirect rates can be charged, as sub-award information is not uniformly available. The potential significance of these exclusions are discussed in the results.

Retention rates developed are then multiplied against *Tot*_*ijt*_ to calculate *Ret*_*ijt*_.

### Other excluded costs (*EC*_*ijt*_)

After excluding sub-award amounts, we subtracted other excluded costs from the remaining sum. For capital expenditures, we exclude COP funding under the “Construction”, “Renovation”, and “Construction/Renovation” cross-cutting categories. As these data only exist from 2010–2016, we applied proportional rates for all partner funding (Total_ijt_) for years 2007–2009.

For equipment costs, we exclude COP funding under the “Motor Vehicles: Purchased” cross-cutting category as well as 40% of all COP funding under the HLAB (Laboratory Infrastructure) budget code. As with capital expenditures, cross-cutting vehicles data are only available after 2013. Therefore, we applied proportional rates to partner funding for preceding years.

For ARV procurement, we exclude all COP funding under the HTXD (ARV Drugs) budget code.

We made no attempt to exclude rental costs as no data on such expenses exist in the COPs.

Combined excluded costs (*EC*_*ijt*_) are then subtracted from *Ret*_*ijt*_ to estimate the non-excludable costs (*NEC*_*ijt*_).

### Calculating indirect costs from *NEC*_*ijt*_

Non-excludable costs (*NEC*_*ijt*_) combines MTDC and indirect costs. To calculate what proportion of *NEC* is indirect costs, it is necessary to know either: 1) Actual MTDC; or 2) the indirect cost rate. Neither are fully publicly available across all partners.

US-based Universities regularly post either their full NICRAs or indirect cost rates online. For all US-based Universities included in our analysis, current or historic NICRAs were publicly available (n = 28). University NICRA agreements have separate rates for different activities; for example, research, instructional activities, or other sponsored activities (OSA). As noted by Johns Hopkins University, OSA includes “health services projects and community service programs”.[23] We assume this practice is consistent across Universities. Rates are also differentiated by whether the activities are considered “on-campus” or “off-campus” depending on where the predominance of the work is being conducted. As our intention is to develop a range, the indirect cost rates for both on-campus and off-campus OSA were recorded. Where historic rates were unavailable, current rates were applied retrospectively.

For IOs and non-US-based Universities, NICRAs are not publicly available to our knowledge. We requested sharing of NICRAs on a confidential basis from the 30 largest IOs by total COP funding, but only three IOs agreed to share their rates. Due to the risk of bias in the low response rate and in order to comply with PlOS data availability requirements, these rates were not included in the results published here.

For IOs and non-US-based Universities where indirect rate data were not available, we applied several scenarios outlined below.

The calculation to get *Ind*_*ijt*_ with a known or assumed indirect cost rate and *NEC*_*ijt*_ is then:

$${Ind}_{ijt}=\frac{{NEC}_{ijt}}{\left(1+r_{ijt}\right)}*r_{ijt}$$

### Rate scenarios

Since indirect cost rates were only known for the 28 partners for which we were able to identify NICRAs, we calculated indirect costs in six potential scenarios described below from least conservative to most conservative. This methodology produces an illustrative range of possible indirect costs accrued within the PEPFAR program.

- Scenario A: Assumes all contracts or grants with universities have indirect rates based on “On-Campus” OSA rates. For IOs, we applied the average of university on-campus rates.
- Scenario B: Assumes all contracts or grants with universities have indirect rates based on “Off-Campus” OSA rates. For IOs, we apply the same methodology as Scenario A.
- Scenario C: For universities, as in Scenario B. For IOs, we applied the average of university off-campus rates.
- Assumed 20% / 15% / 10%: For universities, as in Scenario B. For IOs, we use the given assumed rate (20%, 15%, or 10%). As 10% represents the available *de minimus* rate available to all partners without an established NICRA, the Assumed 10% scenario models a situation where no additional IOs have established NICRAs.

### Validation sources

Finally, since planned funding levels in COPs and actual expenses may not align, FY2012 –FY2015 PEPFAR Expenditure Analysis (EA) data on actual expenditures incurred by implementing partners were compiled from PEPFAR’s website and used to substantiate data in the COPs.[16]

### Data availability

The model was implemented in Python 2.7. The full model and accompanying data are available on GitHub.[24]

## Results

Of $37.01 billion in total COP funding between 2007 and 2016, $15.39 billion (41.59%) was identifiably allocated to IOs, $8.64 billion (23.36%) to local organizations, $3.68 billion (9.95%) to universities, $4.02 billion (10.87%) to USG, and $5.68 billion (14.23%) was NA. Of NA, 48.46% and 11.60% were proportionately allocated to IOs and Universities respectively ($2.55 billion and $0.61 billion). In sum, $22.24 billion (60.08%) of COP funding was included in the model. Exclusions and results are shown in Fig 1.

**Fig 2:**
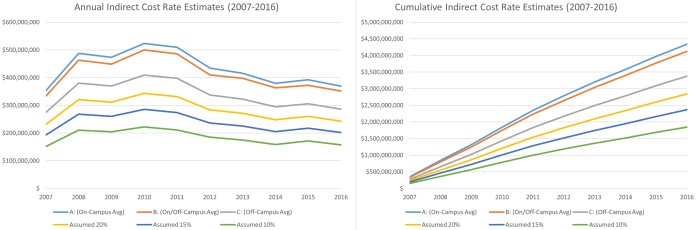
Annual and cumulative range of estimated indirect costs (2007–2016).

From 2007–2016, across the 6 scenarios modeled, we estimate indirect costs of $4.34 billion (19.51%), $4.13 billion (18.55%), and $3.38 billion (15.19%) for Scenarios A, B, and C respectively, and $2.84 billion (12.79%), $2.37 billion (10.64%), and $1.85 billion (8.30%) for the Assumed 20%/15%/10% scenarios (Table 1). From 2007–2016, the range of potentially accrued indirect costs spanned $2.49 billion (11.21%) (Scenario A compared with Assumed 10%). In 2016, the extent of the range was $211.65 million (10.70%) (Fig 2).

**Table 1:** Total COP funding, exclusions, and scenario indirect cost estimates (2007–2016).

In ‘000	2007	2008	2009	2010	2011	2012	2013	2014	2015	2016	Total
Total Funding	1,878,891	2,580,074	2,439,936	2,629,582	2,514,475	2,144,056	2,091,740	1,951,594	2,029,877	1,977,657	22,237,882
Exclusions											
*Sub-Awards*	*(189*,*820)*	*(257*,*571)*	*(240*,*180)*	*(242*,*394)*	*(215*,*096)*	*(166*,*268)*	*(151*,*233)*	*(141*,*020)*	*(150*,*218)*	*(164*,*171)*	*(1*,*917*,*972)*
*Capital Expenditures*	*(15*,*925)*	*(21*,*868)*	*(20*,*680)*	*(21*,*960)*	*(20*,*851)*	*(30*,*868)*	*(22*,*123)*	*(14*,*640)*	*(14*,*285)*	*(5*,*343)*	*(188*,*541)*
*Motor Vehicles*	*(9*,*267)*	*(12*,*725)*	*(12*,*034)*	*(12*,*969)*	*(12*,*402)*	*(10*,*575)*	*(16*,*833)*	*(10*,*783)*	*(10*,*056)*	*(10*,*675)*	*(118*,*319)*
*Lab Equipment*	*(33*,*686)*	*(51*,*924)*	*(55*,*154)*	*(51*,*891)*	*(42*,*857)*	*(42*,*882)*	*(49*,*381)*	*(23*,*272)*	*(20*,*377)*	*(16*,*334)*	*(387*,*759)*
*ARV Drugs*	*(293*,*509)*	*(385*,*787)*	*(315*,*336)*	*(315*,*757)*	*(297*,*154)*	*(254*,*459)*	*(279*,*759)*	*(325*,*836)*	*(337*,*774)*	*(380*,*344)*	*(3*,*185*,*715)*
Total NEC	1,336,684	1,850,198	1,796,552	1,984,611	1,926,116	1,639,005	1,572,411	1,436,043	1,497,166	1,400,791	16,439,576
Indirect Cost Estimates											
A: (On-Campus Avg)	352,687	487,936	473,635	523,194	509,869	434,364	415,951	379,624	392,446	368,987	4,338,694
B: (On/Off-Campus Avg)	333,567	463,036	448,540	499,938	485,966	410,058	397,313	363,278	372,605	351,542	4,125,841
C: (Off-Campus Avg)	274,318	380,170	369,448	409,004	397,857	337,271	322,466	294,823	305,645	286,295	3,377,297
Assumed 20%	231,119	320,532	311,596	343,414	331,761	283,334	271,523	247,796	260,022	242,478	2,843,575
Assumed 15%	193,178	267,869	260,510	285,367	273,964	236,295	225,220	205,052	217,596	201,757	2,366,807
Assumed 10%	151,788	210,418	204,779	222,043	210,911	184,980	174,707	158,423	171,314	157,334	1,846,697

NEC: Non-excludable costs (combined modified total direct costs (MTDC) and indirect costs).

Between 2007 and 2009, the average retention rate for partners with disclosed sub-awards was 83.5%. Sub-awards to other IOs and universities were estimated to account for 30.48% of sub-award totals in 2007. Average agency retention rates are shown in Table 2. Sub-award exclusions totaled $1.92 billion (8.62%).

**Table 2:** Applied partner retention rates by agency. Average retention rates based only on partners reporting subpartners. Average IO Proportion is the proportion of sub-award funding to other international partners. Applied average is the retention rate applied where partner data are lacking.

Funding Agency	Average Retention Rate	Average IO Proportion	Applied Average
HHS/CDC	86.33%	26.75%	89.99%
Dept of Labor	99.17%	0%	99.17%
Dept of Defense	93.97%	76.36%	98.57%
Bur. PRM	93.65%	0.00%	93.65%
HHS/HRSA	68.51%	40.90%	81.39%
USAID	89.06%	28.29%	92.16%
All Others	-	-	87.41%

HHS/CDC: Health and Human Services/Centers for Disease Control and Prevention; Bur. PRM: Bureau of Population, Refugees, and Migration; HHS/HRSA: Health and Human Services/Health Resources and Services Administration; USAID: United States Agency for International Development.

Exclusions for capital expenditures totaled $188.54 million (0.85%). EA data from 2012–2015 show capital expenditures accounting for 1.7% of total spending, suggesting COP data under-report capital expenditures. However, capital expenditures in COPs are disproportionately within USG entities, which are excluded from our model. Total COP capital expenditures across all entities account for 1.1% of total COP funding over that period.

Exclusions for equipment were estimated at $118.32 million (0.53%) for purchased motor vehicles and $387.76 million (1.74%) for laboratory infrastructure. Both amounts are in line with EA data (Vehicles: 0.56%, Equipment and Furniture: 2.05%). ARV drug exclusions were estimated at $3.19 billion (14.33%) compared with EA data for ARV drugs at 10.1% of expenditures, and 14.65% of expenditures including both ARV and “Non-ARV drugs and reagents”. After exclusions, $16.44 billion (73.93%) remained as NEC.

Average applied NICRA rates for IOs were 36.28% and 26.13% (±0.3% by year) for Scenarios A/B and C respectively.

## Discussion

To the best of our knowledge, this analysis is the first to quantify a range of plausible indirect costs among PEPFAR University and IO implementing partners. These entities make up the backbone of PEPFAR service delivery, receiving over 60% of total COP funding in our analysis. The current assessment does not speak directly to whether indirect costs are too low or too high as data on the quantity and quality of services delivered are not available. Nevertheless, our estimated range of PEPFAR funding for indirect costs between $4.34 billion (19.51%) and $1.85 billion (8.30%) amongst these partners between 2007 and 2016 speaks to the difficulty in accessing information on indirect costs as a component of such assessments and represent an opaque layer of programmatic costs. Given the current donor funding environment, re-assessing the current policies and structures for indirect cost recovery is appropriate.

This analysis should not be taken as a critique of the PEPFAR program, which is a model of transparency and demonstrable impact among global health and development programs. This analysis is only possible because detailed PEPFAR financial data are publicly available. As indicated above, PEPFAR itself is bound by the general federal government regulations relating to indirect cost recovery. We know of no legal authority for PEPFAR to directly control the rates agencies are obligated to pay. As such, the responsibility for determining appropriate indirect costs lies with two primary sources: 1) implementing partners; and 2) the US Congress.

Our analysis should also not serve as a general indictment of indirect costs. There is no question that organizations need indirect cost recovery to effectively administer and run programs that save lives. Our analysis is intended to quantify the potential range of indirect costs of IOs and Universities to enable more informed debate regarding potential areas for increased efficiencies. The content of that debate is a question for policy-makers, advocates, financial experts, and implementing partners to develop. We offer a few points of consideration below relating to transparency and program sustainability.

First, however, it is important to note that PEPFAR’s role in LMIC countries cannot be overstated: the program has reduced drug prices through bulk procurement, changed the narrative around the feasibility and affordability of treatment programs in developing countries, and made great strides in HIV prevention. PEPFAR and its implementing partners unlocked the ability to deliver programs, fundamentally changing the course of the HIV/AIDS epidemic and fueling new research and knowledge that have made the current 90-90-90 aspirations achievable.

### Transparency in indirect costs and NICRAs

What is known of indirect cost rates for PEPFAR comes largely from University rates. Aside from US-based Universities, little reliable information is publicly available for partners working in international development. In searches of USAID’s website, limited grant reports and proposals obtained reveal stated or potential indirect rates ranging from 12% to as high as 54%. But these are not systematically available and represent relatively few partners. Of the three NICRA rates that were shared with us confidentially, the average rate was 16.32% with a range between 12.44% and 18.50%. Nevertheless, the broad range of rates and the total amount of funding potentially implicated suggests that different partner structures may be able to implement programming with lower overhead.

That said, it is important to note that the rates that we have identified and modeled are significantly below previously published indirect cost rates for non-profits engaged in medical research (52%-55%).[25, 26] This is not surprising given the different contexts of service delivery programs and clinical research, but does speak to the need for better transparency in the specific area of service delivery. In contrast to PEPFAR—and development programming generally—the National Institutes of Health (NIH) publicly reports both direct and indirect costs through its RePORTER database.[26] Doing so enables a level of insight into the operational costs of NIH funded research that is unlike what is available in the development context.

From a policy perspective, greater transparency should be encouraged to enable better analyses of the return on investment that comes from indirect cost recovery and qualitative assessments of organizational structures that reduce overhead. There are several forms that transparency could take, from directly reporting indirect costs associated with each grant—as in the case of NIH—or amending regulations to enable NICRAs to be publicly available. Currently, NICRAs are considered proprietary information and exempted from disclosure laws. Alternatively, partners could make their NICRAs publicly available as US based universities do, though a voluntary approach could bias research on these data.

### Local programming and sustainability

The NICRA rates negotiated with universities and IOs contrast sharply with rates reimbursed to local organizations. While eligible for establishing NICRAs, many local organizations lack the financial, accounting, and other regulatory requirements necessary to negotiate a NICRA or are not aware of the option.[19] As such, they develop and implement programs where indirect costs are either direct billed or capped at the *de minimus* 10% regulatory limit.[19, 27]

Insufficient capacity of local organizations to manage large grants is often cited to justify the persistently large share of PEPFAR funding to IOs; however, as PEPFAR itself has stated, long-term sustainability requires that local capacity—including management capacity—be fully developed.[28] The role of indirect costs as an investment in such capacity development should not be dismissed. The under-availability or under-utilization of NICRAs by local implementing partners could contribute to a slower process of developing this local capacity—with the subsequent effect of delaying local partners’ from taking over the bulk of service delivery responsibilities.

While there are different decisions and trade-offs to be made between focusing on creating greater local capacity and focusing on rapid scale-up, in many PEPFAR program countries in sub-Saharan Africa, PEPFAR is the largest financer of the HIV response. If long-term program sustainability depends on capacitating local organizations to take over the bulk of programmatic responsibilities currently provided by IOs and Universities, the regulatory hurdles that undermine local organizations accessing sufficient indirect cost recovery should be evaluated and, where possible, addressed.

At the same time, it must be recognized that many local organizations have been able to develop and thrive with support from PEPFAR. Previous analysis of PEPFAR funding identified 122 distinct local partners that evolved from sub-contractors on PEPFAR grants to primary recipients between 2005 and 2015, suggesting positive evolution of capacity development.[29]

### Future data collection and analysis

Our model does not assess the quality, quantity, or unit costs of services being provided by partners. Data regarding program performance by implementing partners have only recently become available from PEPFAR.[17] Even with partner data, however, there may not be sufficient data on program context to make meaningful comparisons of partner performance and efficiency. Better data and analysis regarding the quantity, quality, and specifics of services provided by implementing partners—including details on the contributions of other partners, funders, and domestic governments and measured at the facility or community level—would assist in developing a better understanding of whether investments made into indirect costs translate into higher quality services or greater efficiencies.

## Limitations

Due to uncertainty on the completeness of sub-award data in the COPs, we have made intentionally conservative decisions in calculating sub-award exclusions. EA data do not provide a means for verification of sub-awards. While sub-award data from USAspending.gov could provide a means for verification, at present, it is not possible to link PEPFAR data from the COPs to USAspending data for all agencies.[30] First, our methodology inherently assumes that every contract or grant from PEPFAR has sub-awards. Second, when calculating agency averages, we treated all sub-partners receiving less than $100,000 as local organizations. Third, we made no attempt to include indirect costs on the first $25,000 of each sub-award. Finally, as local organizations were excluded from the model, we do not include modelling of indirect costs incurred when local organizations sub-award to IOs or universities.

Cooperative agreements and grants may include “cost sharing”, either as a required or voluntary contribution to the project that effectively lowers project costs to the USG—including in the form of donated indirect costs. However, data on the prevalence or proportion of indirect costs that may be off-set through cost sharing requirements on PEPFAR grants are not generally available.

Finally, we only model funding allocated through the COP process. Total appropriated bilateral PEPFAR funding from 2007–2016 was $50.4 billion, of which $13.6 billion was not identifiably allocated in COPs.[3] PEPFAR funding for some countries falls below the threshold necessary for creating and submitting a COP. Moreover, planning or expenditure data for centrally funded initiatives are not available in sufficient detail to identify the partners and potential indirect costs associated with such funding. Greater transparency in this area is encouraged.

## Conclusion

While great strides have been made in improving efficiencies, developing programs, and infrastructure to improve outcomes for PLHIV globally, much is yet to be done. In this effort, increasingly limited development dollars necessary for ARV and other commodities necessitates that all potential sources for program efficiency be critically assessed. The policies around indirect cost recovery warrant investigation in such assessments as potential avenues for greater efficiencies—with the goal of identifying whether indirect cost recovery rates bring the return on investment necessary to justify the expenditure and whether differences in cost structures are able to achieve greater efficiencies. Globally, only 53% of PLHIV are accessing treatment—47% are still waiting.[31]
